# Regular Intake of Green Tea Polyphenols Suppresses the Development of Nonmelanoma Skin Cancer through miR-29-Mediated Epigenetic Modifications

**DOI:** 10.3390/jcm11020398

**Published:** 2022-01-13

**Authors:** Vikash Kansal, Anshu Agarwal, Angela Harbour, Humaira Farooqi, Vijay Kumar Singh, Ram Prasad

**Affiliations:** 1 Department of Otolaryngology, Emory University, Atlanta, GA 30322, USA; vikash.kansal@emory.edu; 2 Department of Zoology, Agra College, Agra 282001, India; anshuagarwal112@gmail.com; 3 Department of Biotechnology, Hamdard University, New Delhi 110048, India; 4 College of Medicine, Florida State University, Tallahassee, FL 32304, USA; ah13d@med.fsu.edu; 5 Department of Ophthalmology and Visual Sciences, University of Alabama at Birmingham, Birmingham, AL 35294, USA

**Keywords:** miR-29, DNA methylation, UVB radiation, skin cancer, green tea polyphenols

## Abstract

Previously, we and others have shown that the regular intake of green tea polyphenols (GTPs) reduces ultraviolet B (UVB) radiation-induced skin cancer by targeting multiple signaling pathways, including DNA damage, DNA repair, immunosuppression, and inflammation. Here, we determine the effect of GTPs on UVB-induced epigenetic changes, emphasizing DNA hypermethylation in UV-exposed skin and tumors and their association with miR-29, a key regulator of DNA methyltransferases (DNMTs). Skin cancer was induced in SKH-1 hairless mice following repeated exposures of UVB radiation (180 mJ/cm^2^, three times/week, 24 weeks) with or without GTPs supplementation (0.2%) in drinking water. Regular intake of GTPs inhibited tumor growth by hindering the cascade of DNA hypermethylation events. GTPs supplementation significantly blocked UVB-induced DNA hypermethylation in the skin (up to 35%; *p* < 0.0001) and in tumors (up to 50%; *p* < 0.0001). Experimental results showed that the levels of DNA hypermethylation were higher in GTPs-treated mice than in the control group. The expressions of miR-29a, miR-29b, and miR-29c were markedly decreased in UV-induced skin tumors, and GTPs administration blocked UVB-induced miR-29s depletion. Furthermore, these observations were verified using the in vitro approach in human skin cancer cells (A431) followed by treatment with GTPs or mimics of miR-29c. Increased levels of miR-29 were observed in GTPs-treated A431 cells, resulting in increased TET activity and decreased DNA hypermethylation. In conclusion, UVB-mediated miR-29 depletion promotes DNA hypermethylation and leads to enhanced tumor growth by silencing tumor suppressors. Regular intake of GTPs rescued UVB-induced miR-29 depletion and prevented tumor growth by maintaining reduced DNA hypermethylation and activating tumor suppressors. Our observations suggest that miR-based strategies and regular consumption of GTPs could minimize the risk of UVB-induced skin cancers and contribute to better management of NMSCs.

## 1. Introduction

Exposure of the skin to UVB causes DNA damage, oxidative stress, inflammation, and immune suppression, and ultimately contributes to a wide range of skin diseases, including skin malignancies [1]. Skin cancer is among the most common cancers in the United States [2]. All forms of skin cancer, including basal cell carcinoma (BCC), squamous cell carcinoma (SCC), and melanoma, are known to be linked to chronic UVB exposure. Each year, there are roughly 1.3 million new diagnoses of skin cancer in the United States [3]. The prevalence of skin cancer is equal to the cumulative occurrence of cancers in all other organs, indicating a serious public health issue [4]. Along with acquiring a deeper understanding of disease manifestation at a molecular level, it is also crucial to dive into possible preventions in everyday life that preclude the problem altogether. Due to their anti-inflammatory, anti-oxidative stress, and immunoprotective properties, biologically active phytochemicals are being investigated extensively for their role in prevention and intervention in different diseases. Green tea is one of the most common beverages worldwide, especially in Asian countries such as Japan, Korea, parts of India, China, North Africa, and many Middle Eastern countries [1,2,3,4,5]. Recent research in the United States and other western countries indicates that green tea has health benefits and potent anti-carcinogenic properties for a wide range of malignant pathologies including, but not limited to, lung, prostate, breast, colorectal, melanoma, and skin cancer [6,7,8,9]. Due to the presence of natural phytochemicals, including catechins/epicatechins, green tea is also known as green tea polyphenols (GTPs). Epicatechin-3-gallate (ECG), epigallocatechin (EGC), gallocatechin (GC), and epigallocatechin-3-gallate (EGCG) are the major constituents abundantly found in GTPs [10]. Studies have reported the functional importance of GTPs in the regulation of cancer pathogenesis through inhibiting metastatic potential and cell proliferation by targeting molecular mechanisms including stem cells [6]. Furthermore, GTPs also regulate functional characteristics of human neutrophils via repressing the activation of the TLR-4/NF-κB signaling pathway [11]. GTPs can prevent nonmelanoma skin cancer by boosting DNA repair and inhibiting inflammasome formation and IL-1β secretion, leading to suppression of melanoma skin cancer growth [12].

MicroRNAs (miRs) are small non-coding RNA molecules (17–25 nucleotides) which play a vital role in numerous biological pathways in mammals and other multicellular organisms. A single miR targets up to hundreds of mRNAs. Approximately 30% to 60% of all human genes are impacted by miR regulation. The change in basal levels of miR expression affects several pathologies, including cancer, through modulating cell proliferation, cell cycle, apoptosis, cellular differentiation, migration, and metabolism [13,14], and by acting as an oncogene or tumor-suppressor gene. MiRs repress oncogenic targets as tumor suppressors; however, they are often downregulated in cancer tissues [15], whereas upregulated miRs stimulate cancer progression [16]. One of the fascinating groups of miRNAs is miR-29s, comprising three members: miR-29a, miR-29b, and miR-29c. A depletion of miR-29s expression is reported in carcinogenesis [17]. Investigations have revealed an ameliorating contribution of miR-29s in different pathologies, such as leukemia, melanoma, lung, colon, and cervical cancer for their role in cell proliferation and apoptosis [18,19,20,21,22,23,24]. Further studies have disclosed that miR-29s also directly target DNA methyltransferases (DNMTs) and regulate DNA hypermethylation, a critical epigenetic process implicated in tumorigenesis, development, and promotion.

Here, we investigate the role of GTPs on miR-29-mediated epigenetic modification while focusing on DNA hypermethylation in UVB-exposed skin and tumors. We hypothesized that the oral administration of GTPs may inhibit UVB-induced DNA hypermethylation, DNMT activity, and protein expression in skin and tumors, ultimately leading to the prevention of UVB-induced non-melanoma skin cancer (NMSC). 

## 2. Methods

### 2.1. Antibodies, Chemicals, and Reagents

The primary antibodies specific for DNMT1 (#5032), DNMT3a (#3598), DNMT3b (#57868), 5mC (#28692), Histone H3 (#4499), Sp1 (#9389), Sp3 (#94080), p16 (#80772), p21 (#2947), p27 (#3686), RASSF1A (#86026), TET2 (#36449), and β-actin (#8457) were purchased from cell signaling (Danvers, MA, USA). Antibodies specific for TET1 (#A-1020), TET3 (#A70559), 5hmC (#A-1018), ELISA assay kits for global DNA methylation (#P-1030), DNMT activity (#P-3009), and TET activity (#P-3086-96) were purchased from Epigentek, Inc. (Farmingdale, NY, USA). The secondary horseradish peroxidase antibodies specific for mouse IgG (#sc2748) and rabbit IgG (#sc2357) were purchased from Santa Cruz Biotechnology (Santa Cruz, CA, USA). Both secondary color conjugated antibodies specific for AlexaFluor-488 goat-anti-mouse (#A32723) and AlexaFluor-594 goat-anti-rabbit (#A32740) were purchased from Thermofisher Scientific (Waltham, MA, USA). All other chemicals and reagents of analytical grade were purchased from Sigma-Aldrich (St. Louis, MA, USA).

### 2.2. Green Tea Polyphenols

The purified mixture of GTPs (brand name: Sunphenon 90D, Food grade, purity > 90% polyphenols) was obtained from Taiyo International Inc. (Minneapolis, MN, USA), which contains all major epicatechin derivatives, including (−)-epigallocatechin-3-gallate (EGCG), (−)-epigallocatechin (EGC), (−)-epicatechin-3 gallate (ECG), and (−)-epicatechin (EC). Importantly, we used GTPs in this study to assume that all ingredients may act together additively or synergistically, more than a single constituent. Moreover, GTPs in synergy are more practical as people consume water extracts of green tea as a popular beverage. Experimental mice were given 0.2% GTPs in drinking water. Drinking 0.2% GTPs is equivalent to 10.7 mg/mouse/day, considering an SKH-1 hairless mouse, bodyweight 25.0 gm, drinks 5.35 mL of water [25]. Based on the mouse dose, the human equivalent dose (HED) of GTPs will be approximately 69.03 mg/day, calculated by the formula described by Nair et al. [26].

### 2.3. Cell Line and Culture Conditions

The normal human epidermal keratinocytes (HaCaT) and skin cancer cells (A431), purchased from the American Type Culture Collection (Manassas, VA, USA), were cultured in Dulbecco’s modified Eagle’s medium (DMEM) supplemented with 10% heat-inactivated fetal bovine serum (Hyclone, Logan, UT, USA), 10,000 units/mL penicillin-streptomycin, and in an incubator with 5% CO_2_ at 37 °C. A stock solution of GTPs was prepared in sterile PBS and mixed into the cell culture medium to acquire the desired concentration into sub-confluent cells (60–70% confluent). A431 cells were treated with GTPs in a dose-dependent manner (0, 10, 20 µg/mL) for 5 days. 

### 2.4. Animals

Experimental mice (SKH-1 hairless mice; 6–7 weeks old) were purchased from Charles River Laboratory (Wilmington, MA, USA). The mice were kept for at least one week in our animal resource facility before being used in experiments. Standard conditions of a 12 h dark/12 h light cycle, a temperature of 24 ± 2 °C, and relative humidity of 50 ± 10% were maintained. The experimental protocol for the animal study (APN: 09670) was approved by the Institutional Animal Care and Use Committee (IACUC) at University of Alabama at Birmingham, USA.

### 2.5. UVB Irradiation and Photocarcinogenesis

The mice were exposed to UVB radiation as described previously [27]. Briefly, the dorsal skin of SKH-1 hairless mice was exposed to UVB radiation from a band of four FS24T1 UVB lamps (Daavlin, UVA/UVB Research Irradiation Unit, Bryan, OH, USA) equipped with an electronic controller to regulate UV dosage. Under the standard photocarcinogenesis protocol, mice were UVB irradiated (180 mJ/cm^2^; three times per week) for 24 weeks.

### 2.6. Tissue Collection, miRs Extraction, and RT-PCR

After photocarcinogenesis (24 weeks), mice from all cohorts were euthanized, and skin and tumors were harvested. The tissues were then divided into several fractions for protein estimations, mRNA expression, and immunostaining and stored at appropriate conditions until further analysis. The TRIZOL-chloroform extraction method was used to separate total RNAs, which contained 95 percent miRNAs [28] from HaCaT and A431 cells, skin, and tumors. The iScript cDNA Synthesis Kit (Bio-Rad, Hercules, CA, USA) was used to synthesize cDNA from total RNA, as per the manufacturer’s instructions. Platinum Taq DNA Polymerase (Invitrogen, Carlsbad, CA, USA) kit was used to perform RT-PCR with mouse-specific primers for miR-29a, miR-29b, and miR-29c following the PCR conditions: Stage I: 95 °C for 3 min, 53 °C for 1 min, 72 °C for 30 secs (2 cycles); Stage II: 95 °C for 3 min, 53 °C for 1 min, 72 °C for 30 secs (55 cycles); Stage III: 72 °C for 5 min. Each sample’s PCR product was run on a 2.5 percent agarose gel in 1× Tris-acetate EDTA buffer containing EtBr and examined with a Gel-Doc instrument. The primers specific for miR-29s were purchased from Origene (Rockville, MD, USA), and the details are provided in Supplementary Table S1. 

### 2.7. Detection of 5mC and 5hmC Proteins by Immunofluorescence

To determine the effect of GTPs on the DNA hypermethylation, skin and tumor tissue sections from all cohorts were subjected to double immunofluorescence staining for 5mC and 5hmC. Briefly, tissue sections were fixed in 10% buffered formalin for 4 h and then embedded into paraffin blocks. To avoid the non-specific binding of primary antibodies, sections were blocked with 2% BSA (bovine serum albumin) in phosphate buffer saline (PBS) for 30 min. Primary antibodies specific to 5mC and 5hmC (1/300 dilution) were used to incubate samples overnight at 4 °C. The next day, PBS was used to wash samples to remove unbound primary antibody and then AlexaFluor conjugated secondary antibodies were used to analyze the expression of proteins. 5hmC was detected using goat anti-mouse IgG tagged with green-fluorescent (AlexaFluor488) dye, while 5mC expression was detected using goat anti-rabbit IgG labeled with red-fluorescent (AlexaFluor594) dye. Finally, the samples were mounted using Vectashield mounting media with DAPI fluorescence (#H1200, Vector Laboratories, Burlingame, CA, USA). A fluorescent microscope was used to perform immunofluorescence detection, and representative images were acquired.

### 2.8. Western Blot

Tissue and cell lysates (cytoplasmic and nuclear fractions) were prepared from the skin, tumors, and cultured cells from all cohorts using the cell fractionation kit purchased from Cell Signaling (#9038) following the manufacturer’s instructions. Protease inhibitor was used to avoid protein degradation supplied with the cell fraction kit. Protein content in the samples was measured using a colorimetric BCA protein assay purchased from Thermofisher Scientific (#23225). The proteins (60–80 µg) were resolved using 8–12% SDS-PAGE gels (Bio-Rad, Hercules, CA, USA) and transferred onto a nitrocellulose membrane. Nitrocellulose membranes were then incubated in the blocking buffer (5% BSA in TBST containing 0.05% Tween20) to block the non-specific binding sites. Then, the membrane was incubated with the protein-specific primary antibody (1/500 dilution) overnight at 4 °C. TBST was used to wash the membrane (5 min each, 3 times) to remove excess antibody. The membrane was then incubated with the appropriate peroxidase-conjugated secondary antibody (1/1000 dilution) at room temperature for 2 h while rocking, followed by washing with TBST (5 min each, 3 times). Protein bands were visualized using chemiluminescence reagents. The membrane was stripped and re-probed with β-actin antibody for cytoplasmic proteins and histone H3 for nuclear proteins to confirm equal protein loading. Western blot membranes were split into two or three pieces based on the molecular weight of the proteins and incubated with different antibodies in some situations. Other protein expressions were identified using protein ladders as molecular weight markers.

### 2.9. Dot-Blot Analysis of 5mC for DNA Methylation

Dot-blot analysis of genomic DNA was performed on skin samples as described previously [29]. In brief, a vacuum dot-blot procedure (Bio-Dot Apparatus; Bio-Rad, Hercules, CA, USA) was used to transfer genomic DNA (50 ng) to nitrocellulose membranes before RNA extraction and fixed by baking the membrane at 80 °C for 30 min. Non-specific sites were blocked by using 5% BSA solution by incubating at 4 °C for 1 h. Membranes were then incubated with anti-5mC antibody overnight at 4 °C, washed in TBST buffer, and probed with a secondary antibody conjugated with HRP. The circular bands of 5mC were detected using enhanced chemiluminescence reagents.

### 2.10. Global DNA Methylation, DNMT, and TET Activity

The levels of global DNA methylation, DNMT, and TET activity were measured in the nuclear fractions of A431 cells, skin, and tumors tissues using the commercially available ELISA assay kits following instructions provided by the manufacturer.

### 2.11. Mimic-miR-29c Transfection

The expression of miR-29c in A431 cells was restored by transfecting them with hsa-miR-29c (#HMI0439-5NMOL, sigma) to study functional analysis. Briefly, A431 cells (1 × 10^5^) were seeded into 6-well culture plates. A431 cells were transfected in serum-free media with the mimic-miR-29c (100 nM) or scramble control probe using Lipofectamine 2000 (Invitrogen) according to the manufacturer’s instructions at 60–70% confluency. After 24 h of transfection, cells were supplemented with 2% FBS in a culture medium. After 48 h, cells were harvested and used in the functional assay by measuring DNA methylation, DNMT, and TET activity. For the transfected A431 cells, both untransfected and scramble probes were utilized as controls.

### 2.12. Statistical Analysis

The statistically significant differences between experimental cohorts were analyzed using one-way ANOVA (Tukey’s multiple comparisons test) through GraphPad Software (Version 9; San Diego, CA, USA). A *p*-value < 0.05 was considered significant.

## 3. Results

### 3.1. GTPs Inhibit Global DNA Methylation and DNMT Activity in SKH-1 Hairless Mice

**Figure 1:**
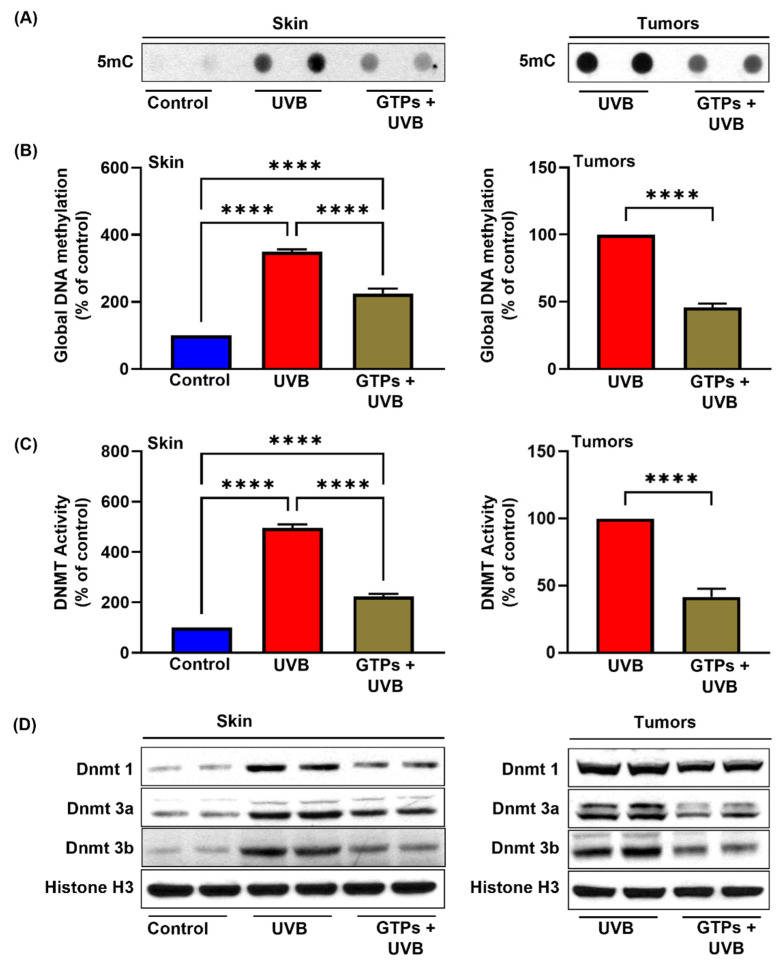
Effect of GTPs on global DNA methylation, DNMT activity, and protein expression in skin and tumors of SKH-1 hairless mice. At the end of the experiment, tumors and skin were harvested after euthanization of mice and subjected to DNA isolation for analysis of DNA methylation by dot blot and ELISA (**A**,**B**). Nuclear fractions were prepared and the effect of GTPs was observed on DNMT activity and protein expression (**C**,**D**). Data are presented as a percentage of control (assigned as 100%) ± S.E.M. Each column represents a pool of three samples obtained from three different mice (*n* = 6). Significant inhibition between UVB alone vs. UVB + GTPs, **** *p* < 0.0001.

DNA hypermethylation is one of the fundamental epigenetic mechanisms that control gene expression and contributes to pathogenesis by silencing the tumor suppressors. Therefore, to determine the effect of GTPs on UVB-induced DNA hypermethylation, we measured DNA methylation levels using dot blot analysis, ELISA, and Western blots. As seen in Figure 1A,B, the expression of 5mC and the levels of global DNA methylation were higher in UVB-exposed skin (3.5-fold; *p* < 0.0001) compared with non-UVB exposed skin (control). Oral administration of GTPs in drinking water reduced UVB-mediated DNA hypermethylation in the skin (225.2 ± 14.41 vs. 349.0 ± 7.43; *p* < 0.0001) and in tumors (up to 50%; *p* < 0.0001). However, the levels of DNA hypermethylation were higher in GTPs-treated skin compared with the control group.

The addition of the methyl group to the fifth position of the cytosine ring, known as DNA hypermethylation, is regulated by DNA methyltransferases (DNMTs). Therefore, we measured the effect of GTPs on DNMT activity. Increased DNMT activity was observed in UVB-irradiated skin (5.0-fold; *p* < 0.0001) compared to the control skin (Figure 1C). GTP treatment inhibited UVB-induced DNMT activity in skin (223.3 ± 10.86 vs. 495.1 ± 14.6; *p* < 0.0001) and in tumors (up to 60%; *p* < 0.0001). We also confirmed these results by DNMT1, DNMT3a, and DNMT3b protein expression. Similar to DNA hypermethylation and DNMT activity, all three DNMTs’ protein expression was reduced in GTPs-treated UVB-exposed skin and tumors (Figure 1D).

### 3.2. Effect of GTPs on Sp1 and Sp3 Expression

**Figure 2:**
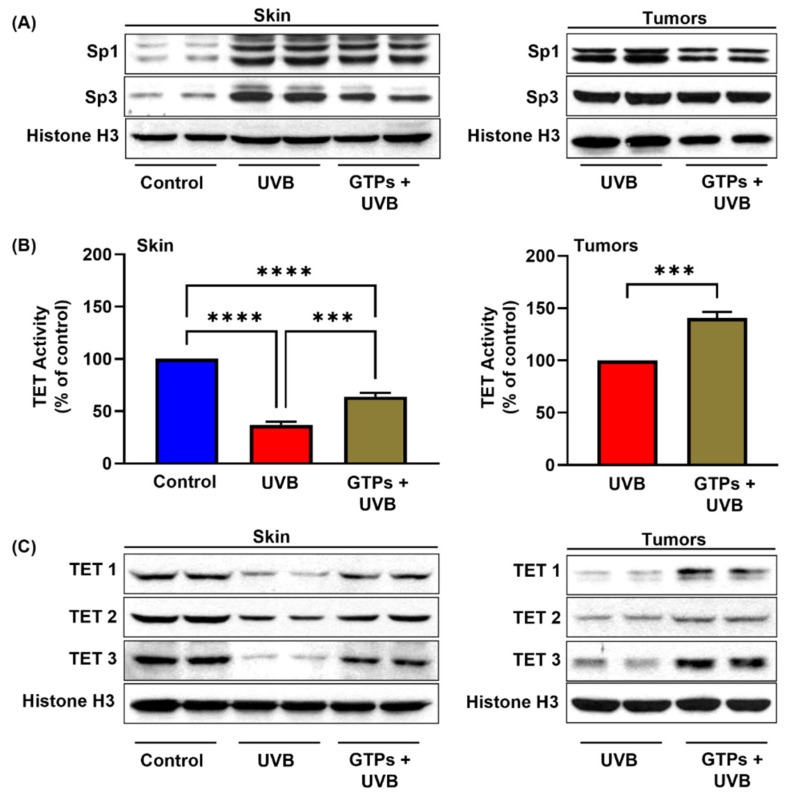
Effect of GTPs on transcription factors, TET activity, and TET protein expression in skin and tumors of SKH-1 hairless mice. After 24 weeks, all mice were euthanized, and tumors and skin were harvested. Nuclear lysates were prepared and subjected to Western blot analysis for the expression level of transcription factors (Sp1 and Sp3) (**A**). Effect of GTPs on TET activity (**B**) and TET 1, TET 2, and TET 3 protein expression (**C**). Data are presented as a percentage of control (assigned as 100%) ± S.D. In the Western blot, each column represents a pool of three samples obtained from three different mice (*n* = 6). Significant increase between UVB alone vs. UVB + GTPs, *** *p* < 0.0002, **** *p* < 0.0001.

Sp1 and Sp3, expressed in all mammalian cells, are transcription factors which regulate protein expression through their binding affinity with DNA [30]. Studies have reported that Sp1 and Sp3 could synergistically activate DNMT1, DNMT3a, and DNMT3b [29,31,32]. Therefore, we deemed it necessary to determine the effect of GTPs on UVB-induced Sp1 and Sp3 expression. The expression of both transcription factors was increased in skin and tumors, while GTPs treatment blocked the influence of UVB on Sp1 and Sp3 activation (Figure 2A). 

### 3.3. GTPs Promote DNA Demethylation

Ten-eleven translocation (TET) enzyme belongs to the family of dioxygenases and has three isoforms: TET1, TET2, and TET3. Several studies have demonstrated that conversion of 5-methylcytosine (5mC) to 5-hydroxymethylcytosine (5hmC) is regulated by TET proteins [33], suggesting a vital role in the development of various diseases, including inflammation, myeloid malignancies, and tumors [34,35,36,37]. Here, we studied the effect of GTPs on TET activity and protein expression. The TET activity in UVB-exposed skin was reduced significantly by up to 63% (*p* < 0.000) compared with normal skin. Our results revealed that GTPs prevent the UVB-induced reduction of TET activity in skin by up to 27% (*p* < 0.0002). TET activity was also observed to be up to 40% higher in GTPs-treated tumors (*p* < 0.0004) than tumors without GTPs (Figure 2B). Loss of UVB-induced TET protein expression was mitigated by GTPs treatment in the skin and tumors (Figure 2C). 

**Figure 3:**
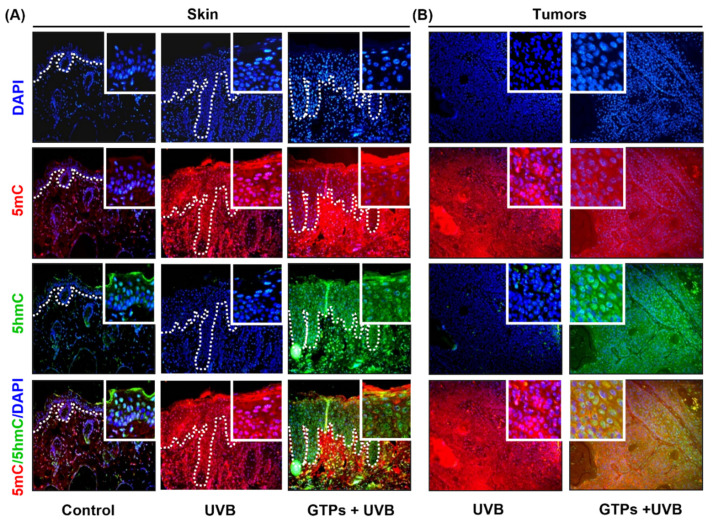
Effect of GTPs on the expression levels of 5mC and 5hmC in UVB-exposed skin and tumors of SKH-1 hairless mice. To define the occurrence of the UVB-induced tumor, the epidermis is demarcated by the dotted white line. The expression of 5mC (red), 5hmC (green), and colocalization (yellow) in the skin (**A**) and tumors (**B**) (*n* = 6).

UVB radiation penetrates the outmost layer of skin (epidermis) and causes maximum damage, leading to skin cancer development [5]. Therefore, next, we confirmed our results by colocalization of 5mC and 5-hmC staining in the epidermis as demarcated by dotted white lines (Figure 3A). Our immunostaining data suggested that the intensity of 5mC-positive cells was higher in UVB-exposed skin and tumors when compared with GTPs-treated cohorts (Figure 3A,B). The expression of 5-hmC was higher in GTPs-treated samples than UVB-exposed skin and tumors. These results suggest that GTPs administration was able to block UVB-induced DNA hypermethylation through activation of DNA demethylation machinery. 

### 3.4. GTPs Treatment Prevents UVB-Induced Depletion of miR-29 Expression

**Figure 4:**
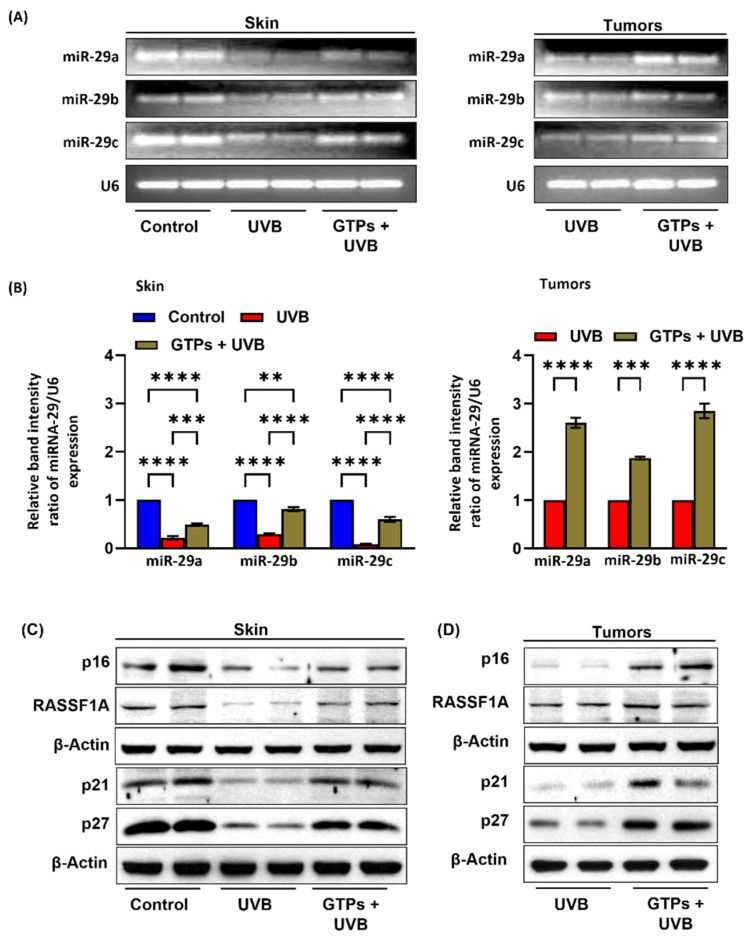
Effect of GTPs on the expression levels of the family members of miR-29 in skin and tumors of SKH-1 hairless mice. Expression levels of miR-29a, 29b, and 29c in skin and tumors (**A**). Relative band intensity of miR-29 expression in skin (left panel) and tumors (right panel), mean values ± S.E.M (**B**). Effect of GTPs on tumor suppressor proteins expression in the skin (**C**) and tumors (**D**) of SKH-1 hairless mice (*n* = 6). U6 was used as a loading control. ** *p* < 0.001 *** *p* < 0.0005, **** *p* < 0.0001.

Next, we determined whether GTPs administration prevents UVB-induced depletion of miR-29 expression in skin and tumors. We observed that the expression of all members of the miR-29 family was reduced upon UVB exposure in skin and tumors (Figure 4A). Our quantification data of band intensity suggest that the expression levels of miR-29a and miR-29b were reduced by 79% (*p* < 0.008) and 71% (*p* < 0.002), respectively. However, the reduction in miR-29c was greater after UVB exposure, at 92% (*p* < 0.002) in UVB-exposed skin compared with control mice (Figure 4B; left panel). GTPs treatment blocks UVB-induced reduction of miR-29a (0.49 ± 0.02 vs. 0.21 ± 0.03; *p* < 0.01), miR-29b (0.81 ± 0.04 vs. 0.29 ± 0.02; *p* < 0.005), and miR-29c (0.60 ± 0.05 vs. 0.08 ± 0.01; *p* < 0.009) in the skin. In the tumors, the expression of these miR-29s remained significantly higher in GTPs-treated UVB-exposed tumors than in the control group (Figure 4B; right panel). Comparisons between groups were made by assigning control skin and UVB tumors an arbitrary value of 1.

### 3.5. GTPs Supplementation Prevents UVB-Induced Loss of Tumor Suppressor Proteins

Tumor suppressor genes synthesize proteins that control cell proliferation and regulate tumor growth. It has been suggested that epigenetic silencing of tumor suppressor genes p16, p21, p27, and RASSF1A is also associated with DNA hypermethylation [38,39,40,41] and contributes to cancer progression. Therefore, we determine the effect of GTPs on p16, p21, p27, and RASSF1A protein expression in skin (Figure 4C) and tumors (Figure 4D). Protein expression of p16, RASSF1A, p21, and p27 was decreased in both UVB-exposed skin and tumor samples as determined by the Western blot analysis. As a result, when exposed to GTPs, the expression levels of p16, RASSF1A, p21, p27 remained higher. 

### 3.6. Reduced Expression of miR-29s in A431 Cells

**Figure 5:**
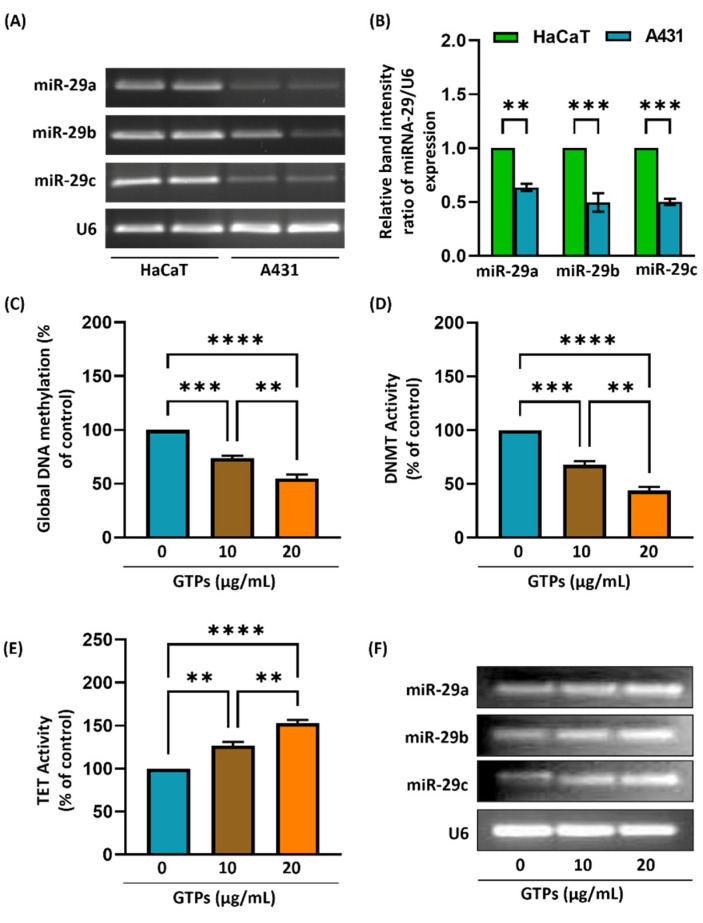
Expression of miR-29 in A431 and the effect of GTPs on epigenetic modulators and miR-29 family members in skin cancer cells. MiR-29 expression in A431 and HaCaT cells (**A**,**B**). Skin cancer cells (A431) were treated with GTPs in dose-dependent manner (0, 10, 20 µg/mL) for 5 days. After 5 days of GTPs treatment, the levels of global DNA methylation (**C**), DNMT activity (**D**), and TET activity (**E**) were measured in all groups. Effect of GTPs on miR-29a, 29b, and 29c (**F**). U6 was used as a loading control. Data are presented as a percentage of the control (non-GTPs-treated) group, which was assigned a value of 100%, and as means ± S.E.M, *n* = 3. ** *p* < 0.002, *** *p* < 0.0009, **** *p* < 0.0001.

To explore the expression levels of miR-29s, we examined the levels of miR-29a, 29b, and 29c in A431 and HaCaT cells using qRT-PCR. As shown in Figure 5A, the human skin cancer cells (A431) express significantly lower levels of all three miR-29 than HaCaT cells. In general, the expression level of miR-29c was lowest among all three miR-29s, as estimated by densitometry quantification of the band intensity using ImageJ software (Figure 5B). 

### 3.7. Effect of GTPs Treatment on DNA Hypermethylation in A431 Cells

To further confirm our observations, we conducted in vitro studies using human skin cancer cells (A431). A431 cells were treated with GTPs in a dose-dependent manner (10 and 20 µg/mL) for 5 days, and the levels of global DNA methylation, DNMT, and TET activity were measured in the nuclear lysates by ELISA. After GTPs treatment with 10 and 20 µg/mL, the levels of DNA methylation were significantly reduced by 26% and 45% (*p* < 0.0009, *p* < 0.0001), respectively (Figure 5C). GTPs treatment also inhibited DNMT activity by 32% and 66% (Figure 5D). These results suggest that GTPs treatment inhibits DNA hypermethylation. Next, we also measured the TET activity in A431 cells. As shown in Figure 5E, GTPs treatment promotes DNA demethylation by 26% and 53% (*p* < 0.002, *p* < 0.0001), respectively. These observations suggest that GTPs treatment not only blocks DNA hypermethylation by inhibiting the addition of a methyl group but also enhances DNA demethylation by converting existing 5mC into 5hmC via TET regulation. 

We also determined the effect of GTPs treatment on miR-29s expression. The expression of all three miR-29s family members was increased in a dose-dependent manner (Figure 5F). Moreover, the effect of GTPs treatment on miR-29c was higher compared to miR-29a and miR-29b.

### 3.8. Effect of miR-29c Mimic on DNA Methylation in A431 Cells

**Figure 6:**
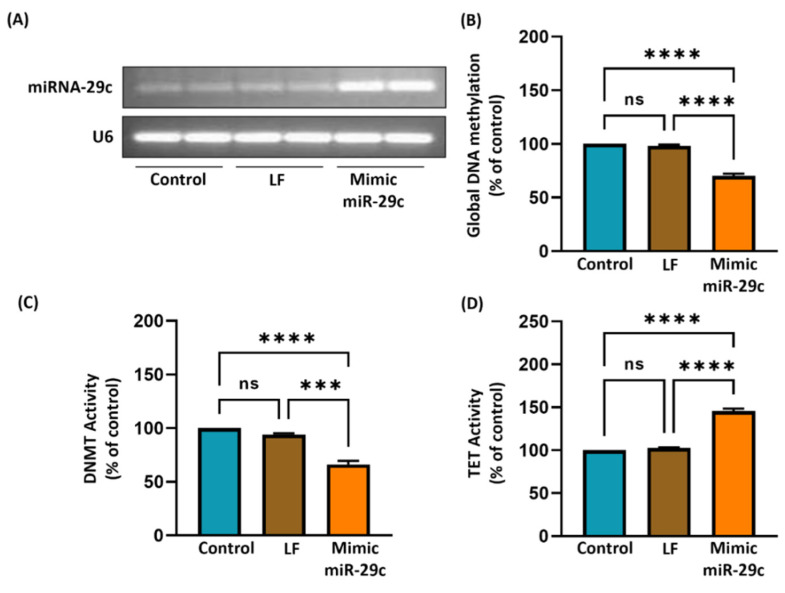
Effect of mimic of miR-29c on epigenetic modulators in skin cancer cells. Skin cancer cells (A431) were transfected with mimic miR-29c (100 nM) in lipofectamine medium for 72 h. (**A**) After 72 h of transfection, miRs were isolated by the Trizol-chloroform extraction method, and cDNA was subjected to RT-PCR using specific primers for miR-29c. U6 was used as a loading control. The levels of global DNA methylation (**B**), DNMT activity (**C**), and TET activity (**D**) were measured in all groups. Data are presented as a percentage of the control (non-transfected) group, which was assigned a value of 100%, and as means ± S.E.M, *n* = 3. LF; Lipofectamine. *** *p* < 0.0002, **** *p* < 0.0001. ns = not significant.

As shown in both in vivo and in vitro studies, the expression of all three miR-29s was altered significantly in UVB-exposed skin and tumors and also in A431 cells. The changes in miR-29c were prominent. Therefore, we investigated the effect of the mimic of miR-29s on DNA methylation. The impact of the mimic-miR-29c on miR-29c expression in A431 cells was confirmed by RT-PCR (Figure 6A). The mimic transfection enhanced miR-29 expression tremendously. The mimic-miR-29c treatment also decreased global DNA methylation by 30% (*p* < 0.0001), DNMT activity by 34% (*p* < 0.0001), and promoted TET activity by 45% (*p* < 0.0001) compared with untransfected or LF-only transfected cells (Figure 6B–D). These results provide a strong indication of the role of GTPs in upregulating miR29 family members and their further role in DNA demethylation.

## 4. Discussion

Prolonged exposure of UVB radiation is one of the critical factors in the onset of skin cancers. NMSCs are one of the most common malignancies predominantly found in Caucasian populations. The United States holds the highest number of cases at an estimated 3 million NMSCs per year [42]. Chemoprevention of NMSCs by natural products has long been considered a promising therapeutic intervention in controlling disease onset and progression, including the use of various botanical agents for the treatment of NMSCs [43]. 

Previous studies have investigated topical GTPs as a treatment for skin cancer [44,45], with significant results in NMSCs. In one particular study, topical treatments rich in GTPs (epigallocatechin gallate) prevented the progression of actinic keratosis to its malignant form of squamous cell carcinoma [46]. This study included 12 weeks of topical epigallocatechin gallate and significantly decreased the severity grade of actinic keratosis. However, the study did not demonstrate agent efficacy due to limitations in compound bioavailability, thus warranting further investigation. Moreover, the results for topical treatment of skin cancers are partly unappreciated in general validity, as GTPs are usually ingested in the form of a beverage. Thereby, as a reputable source of general validity, orally ingested GTPs are under investigation for their role in skin cancer treatment and prevention. Evidence from the literature shows that the role of dietary polyphenols is believed to be important in cancer chemoprevention, as polyphenols have been found to influence the deregulated cellular pathways [47,48,49,50]. Additionally, these dietary factors have also been found to regulate epigenetic and miR mechanisms to confer their anticancer properties. Many of these cancer-associated miRs act as tumor suppressors or pro-oncogenic factors that directly impact cancer progression and metastasis [51]. Earlier studies have revealed the influential role of miR-29 in the repression of DNA methyltransferases [52]. MiR-29 has been found to be a suitable molecular target of dietary agents such as polyphenols [53,54], and modifications of miRs (specifically miR-29) can be directly linked to these dietary polyphenols present in green tea beverages. Studies show that different concentrations of EGCG impart significant results in decreasing hypermethylation in several cancers such as oral, prostate, lung, and breast [55,56,57].

**Figure 7:**
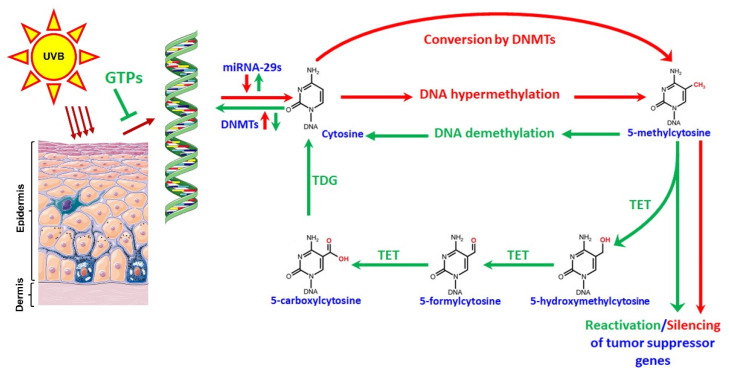
An illustration of the mechanism GTPs adopt to prevent photocarcinogenesis via targeting miR-29s-mediated DNA hypermethylation. Exposure to UVB radiation diminishes the expression levels of miR-29s and leads to activation of DNMTs and promotes DNA hypermethylation, resulting in the silencing of tumor suppressor genes and contributing to tumor growth. GTPs treatment blocks the UVB-induced damage and restores the expression of miR-29s to stop DNA hypermethylation through inhibition of DNMTs. As a parallel mechanism, GTPs treatment activates TET enzymes and converts existing 5mC into 5-hmC followed by 5fC and 5caC to promote DNA demethylation, which leads to growth inhibition by reactivation of silenced tumor suppressors. Red arrows indicate the UVB radiation-regulated pathway while green arrows show the GTPs-regulated pathway. DNMT, DNA methyltransferases; TET, ten-eleven-translocation; TDG, thymine DNA glycosylase. Parts of the figure were obtained from Servier Medical Arts, freely available at https://smart.servier.com.

To expand the literature on the effectiveness of dietary supplements in their role in protecting or treating NMSCs, we assessed the implications that oral GTPs play on the prevention of photocarcinogenesis in irradiated mice and a skin cancer cell line (A431) through epigenetic modifications. The expression of miR-29 was chosen as an investigative target due in part to its role in suppressing DNA methylation and promoting DNA repair as seen in previous studies. The members of the miR-29 family directly target two important factors: DNA methyltransferases and TET enzymes, both involved in DNA demethylation. We observed that the levels of miR-29 family members were downregulated upon UVB radiation, and GTPs administration to mice restored expression of miR-29 and promoted DNA demethylation. We also confirmed our findings in the A431 human skin cancer cell line after GTPs treatment (10 and 20 µg/mL for 5 days), where GTPs treatment upregulated miR-29 expression to promote DNA demethylation and significantly decreased DNMT activity and increased TET activity, further strengthening our hypothesis of the influential role GTPs play in the epigenetic modifications involved in photocarcinogenesis. Together, our findings suggest that GTPs target miR-29 expression to suppress tumorigenesis by promoting changes against existing DNA hypermethylation in a murine model of UVB-induced skin cancer (Figure 7). This study lays key foundation concepts involving the power of GTPs and their ability to modify genetic changes involved in NMSCs in both animal models and skin cancer cell lines; however, further detailed studies are warranted in clinical settings to effectively understand the complete mechanisms involved in the prevention of photocarcinogenesis via epigenetic modifications by polyphenols.

## Supplementary Materials

The following supporting information can be downloaded at: https://www.mdpi.com/article/10.3390/jcm11020398/s1. Table S1: A list of miRNAs primers.
