# Supplementary material for: Regular Intake of Green Tea Polyphenols Suppresses the Development of Nonmelanoma Skin Cancer through miR-29-Mediated Epigenetic Modifications
Source: J Clin Med. 2022 Jan 13;11(2):398. doi: 10.3390/jcm11020398 (PMC8777720; doi:10.3390/jcm11020398)
Supplement: Supplementary file 1 [file jcm-11-00398-s001.zip › jcm-1501699-supplementary.pdf]

Supplementary

Table S1: A list of miRNAs primer's.

miRNA name	Accession number	Forward Primer	Reverse Primer	Catalog No.
mmu-miR-29a	MIMAT0004631	CTGATTTCTTTTGGTGTTCAG	GAACATGTCTGCGTATCTC	MP300235
mmu-miR-29b	MIMAT0000127	AGCACCATTTGAAATCAGTG	GAACATGTCTGCGTATCTC	MP300237
mmu-miR-29c	MIMAT0004632	GACCGATTTCTCCTGGTG	GAACATGTCTGCGTATCTC	MP300238
hsa-mir-29a	MIMAT0004503	CTGATTTCTTTTGGTGTTC	GAACATGTCTGCGTATCTC	HP300281
hsa-miR-29b	MIMAT0004515	GGTTTCACATGGTGGCT	GAACATGTCTGCGTATCTC	HP300283
hsa-miR-29c	MIMAT0004673	TGACCGATTTCTCCTGG	GAACATGTCTGCGTATCTC	HP300284
U6		CTCGCTTCGGCAGCACA	AACGCTTCACGAATTTGCGT	
